# Fault diagnosis method of bearing utilizing GLCM and MBASA-based KELM

**DOI:** 10.1038/s41598-022-19209-1

**Published:** 2022-10-17

**Authors:** Sheng-wei Fei, Ying-zhe Liu

**Affiliations:** College of Mechanical Engineering, Donghua University, Shanghai, 201620 China

**Keywords:** Engineering, Mathematics and computing

## Abstract

In this study, fault diagnosis method of bearing utilizing gray level co-occurrence matrix (GLCM) and multi-beetles antennae search algorithm (MBASA)-based kernel extreme learning machine (KELM) is presented. In the proposed method, feature extraction of time–frequency image based on GLCM is proposed to extract the features of the bearing vibration signal, and multi-beetles antennae search algorithm-based KELM (MBASA-KELM) is presented to recognize the states of bearing. KELM employs the kernel-based framework, which has better generalization than traditional extreme learning machine, and it is necessary to look for an excellent optimization algorithm to select appropriate regularization parameter and kernel parameter of the KELM model because these parameters of the KELM model can affect its performance. As traditional beetle antennae search algorithm only employs one beetle, which is difficult to find the optimal parameters when the ranges of the parameters to be optimized are wide, multi-beetles antennae search algorithm (MBASA) employing multi-beetles is presented to select the regularization parameter and kernel parameter of KELM. The experimental results demonstrate that MBASA-KELM has stronger fault diagnosis ability for bearing than LSSVM, and KNN.

## Introduction

It is well known that the reliable fault diagnosis method for bearing is the key to ensuring the normal operation of equipments^1–3^. It is very significant to study the feature extraction method of the bearing vibration signal due to the important role of the excellent features of the bearing vibration signal for obtaining excellent diagnosis results of bearing^4^. Feature extraction based on time–frequency image of the vibration signal is a popular and effective feature extraction method^5,6^. In this study, short-time Fourier transform (STFT) is used to convert the bearing vibration signal of each sample into the corresponding time–frequency image, and feature extraction of time–frequency image based on gray level co-occurrence matrix (GLCM) is proposed. GLCM is a texture feature extraction method for image pixel processing, which is to count the number of pairs of pixels whose distance is specified in the specified direction. The four features including contrast, correlation, homogeneity, energy based on GLCM are unrelated, which are generally used for classification. However, the four features calculated from gray level co-occurrence matrix of time–frequency image do not achieve good classification effect in the experiment. Therefore, the way of feature extraction needs to be changed.

The excellent classification method is also important to obtain excellent diagnosis results of bearing. K-nearest neighbors (KNN) algorithm is a reliable and simple classification method^7^, which can simultaneously to recognize the bearing's states. Support vector machine (SVM) classifier can solve the classification problems with high dimensions, small training samples, and nonlinearity^8^. Least squares support vector machine (LSSVM) is the least squares version of SVM, which can simplify the training process of SVM and make SVM more practical in engineering applications^9,10^. Kernel extreme learning machine (KELM) generalizes traditional extreme learning machine (ELM) to the kernel-based framework, which has better generalization than traditional ELM^11,12^.

Hereto, fault diagnosis of bearing utilizing GLCM and multi-beetles antennae search algorithm (MBASA)-based KELM is presented in this study, feature extraction of time–frequency image based on GLCM is proposed to extract the features of the bearing vibration signal, and multi-beetles antennae search algorithm-based KELM (MBASA-KELM) is presented to recognize the states of bearing. The regularization parameter and kernel parameter of the KELM model can affect the performance of KELM, so it is necessary to look for an excellent optimization algorithm to select appropriate regularization parameter and kernel parameter of the KELM model. Traditional beetle antennae search algorithm only employs one beetle, one beetle antennae search is difficult to find the optimal parameters when the ranges of the parameters to be optimized are wide. Thus, MBASA employing multi-beetles is presented to select the regularization parameter and kernel parameter of KELM. Multi-beetles antennae search increases the possibility of obtaining the optimal parameters compared with one beetle antennae search. LSSVM and KNN are respectively used to compare with MBASA-KELM. The experimental results demonstrate that fault diagnosis ability of bearing of MBASA-KELM is better than that of LSSVM, and KNN.

Firstly, fault diagnosis method of bearing based on GLCM and MBASA-based KELM is introduced. Then, experimental analysis is performed to testify the superiority of the proposed method. Finally, conclusion is introduced.

## Fault diagnosis method of bearing based on GLCM and MBASA-based KELM

### Feature extraction of time–frequency image based on GLCM

GLCM is a texture feature extraction method for image pixel processing^13^. Its basic principle is to count the number of pixel pairs in a specific direction and distance. Direction is generally divided into four directions: 0°, 45°, 90°and 135°. Distance is a positive integer, which can be manually set to generate a gray level co-occurrence matrix. In order to reduce the amount of calculation, the time–frequency images are processed by changing the image size and gray level.

**Figure 1:**
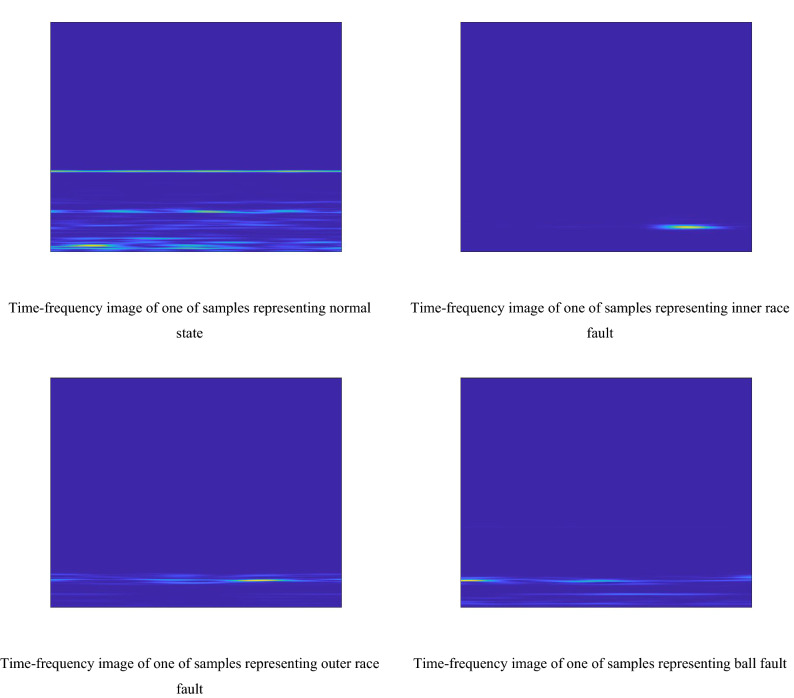
The time–frequency images of a set of samples representing four kinds of state types of bearing.

STFT is used to convert the bearing vibration signal of each sample into the corresponding time–frequency image, and Fig. 1 shows the time–frequency images of a set of samples representing four kinds of state types of bearing. As the time–frequency image generated by STFT^14^ has a large size which is 875 × 656, the size of the image is reduced to 128 × 128. In addition, as the gray image has 256 gray levels, which requires a lot of calculation, the gray level of time–frequency image is set to 4, and the 4×4 gray level co-occurrence matrix is obtained by counting the number of pixel pairs whose direction is 90 degree and whose distance is 2.

The four features including contrast, correlation, homogeneity, energy calculated from gray level co-occurrence matrix of time–frequency image do not achieve good classification effect in the experiment. Therefore, in this study, the $4 \times 4$ co-occurrence matrix is converted into a 16-dimensional vector, which is the preliminary feature of each time–frequency image. Due to the large numerical span of each preliminary feature in the vector, the preliminary features are processed to avoid the impact of too large numerical span on the classification effect. The mapminmax function in MATLAB shown in Eq. (1) is used to normalize the preliminary features in the training set. As mapminmax function is to normalize each row of data, the matrix composed of the preliminary features in the training set is transformed into a row vector before normalization, and then returned to the form of matrix after normalization. When Eq. (1) is used to normalize the preliminary features in the testing set, the values of x_max_ and x_min_ are the maximum and minimum values among the preliminary features in the training set rather than the testing set.

1 $${\text{y = (y}}_{{{\text{max}}}} {\text{ - y}}_{{{\text{min}}}} {)} \times {\text{(x - x}}_{{{\text{min}}}} {\text{)/(x}}_{{{\text{max}}}} {\text{ - x}}_{{{\text{min}}}} {\text{) + y}}_{{{\text{min}}}}$$

where x_max_ and x_min_ are the maximum and minimum values of each row before normalization, respectively, and y_max_ and y_min_ are the maximum and minimum values of each row after normalization, respectively.

The specific steps of extracting the features of time-frequency image based on GLCM are as follows:

Step 1: Reduce the size of time–frequency image from 875 × 656 to 128 × 128.
Step 2: Convert the color image into gray image. At this time, the pixel range of time–frequency image is 0–255.
Step 3: Convert the gray image into an image with four gray levels. At this time, the pixel range of time-frequency image is 1–4.
Step 4: Count the number of pixel pairs whose direction is 90 degree and whose distance is 2, and generate the $4 \times 4$ gray level co-occurrence matrix.
Step 5: Convert the $4 \times 4$ matrix to a 16-dimensional vector, and take this vector as the preliminary feature of time-frequency image. The preliminary features in the training set and testing set are normalized, respectively, and then the features of time-frequency image based on GLCM can be obtained.

**Figure 2:**
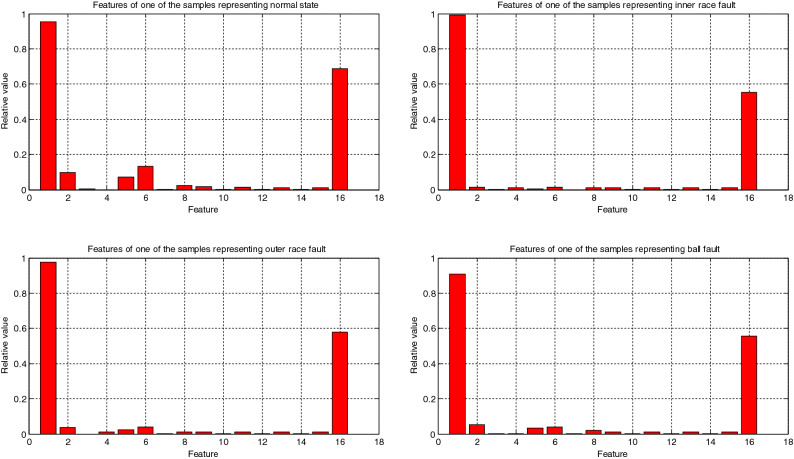
The features of a set of samples with different states by using feature extraction method of time–frequency image based on GLCM.

The features of a set of samples with different states by using feature extraction method of time–frequency image based on GLCM are given in Fig. 2.

### MBASA-based KELM

#### KELM

The detailed description of ELM can be shown in the related literatures^15,16^, ELM can be expressed as follows:

2 $$f(x) = g(x)\omega = g(x)G^{T} \left( {\frac{I}{C} + GG^{T} } \right)^{ - 1} T$$

where $\omega = G^{T} \left( {\frac{I}{C} + GG^{T} } \right)^{ - 1} T$ denotes the weight connecting the hidden layer with the output layer, and *C* denotes the regularization parameter.

KELM employs a kernel function to replace the feature mapping $g(x)$ of ELM, which makes KELM have better convergence and generalization performance than ELM^17^. KELM can be expressed as follows:

$$f(x) = \left[ {\begin{array}{*{20}c} {K(x,x_{1} )} \\ \vdots \\ {K(x,x_{N} )} \\ \end{array} } \right]\left( {\frac{I}{C} + GG^{T} } \right)^{ - 1} T$$

3 $$= \left[ {\begin{array}{*{20}c} {K(x,x_{1} )} \\ \vdots \\ {K(x,x_{N} )} \\ \end{array} } \right]\left( {\frac{I}{C} + \Delta_{KELM} } \right)^{ - 1} T$$

where $\Delta_{KELM}$ denotes the kernel matrix,

4 $$\Delta_{{KELM_{i,j} }} = K(x_{i} ,x_{j} ) = g(x_{i} ) \cdot g(x_{j} )$$

Cauchy kernel is an excellent alternative kernel function, which is employed in this study and can be expressed in the following form:

5 $$K_{Cauchy} ({\mathbf{x}}_{i} ,{\mathbf{x}}_{j} ) = \frac{1}{{1 + \frac{{\left\| {{\mathbf{x}}_{i} - {\mathbf{x}}_{j} } \right\|^{2} }}{\eta }}}$$

where $\eta$ is the Cauchy kernel parameter.

The regularization parameter and kernel parameter of the KELM model can affect the performance of KELM, which need to be carefully selected.

#### Parameter optimization of KELM based on MBASA

BASA imitates the activities of the beetle’s antennae in nature^18^. However, traditional beetle antennae search algorithm only employs one beetle, one beetle antennae search is difficult to find the optimal parameters when the ranges of the parameters to be optimized are wide. Thus, MBASA which employs multi-beetles is presented in this paper. Multi-beetles antennae search increases the possibility of obtaining the optimal parameters.

The process of the parameters' selection of KELM based on MBASA can be described as follows:

Step 1: Define the beetles' positions as a vector ${\mathbf{x}}^{t}$ at the *t*th time instant ( $t = 1,2, \cdots$) . Initialize the parameters of MBASA, including the beetles' position ${\mathbf{x}}^{0}$, antennae length $d^{0}$ and step size $\delta^{0}$.
Step 2: Evaluate the fitness of each beetle fivefold cross-validation method is employed to evaluate the fitnesses of the beetles. In fivefold cross-validation method, the training samples are equally divided into 5 subsets of the samples, among which 4 subsets of the samples are used to train the KELM model, and the remaining subset is used to test the KELM model. Each subset can be used as the testing subset in turn. Then, the total diagnosis accuracy $A_{i}^{{}}$ of the 5 subsets of the samples can be obtained as follows:
  6 $$A_{i} = \frac{{N_{correct,i} }}{{N_{total} }}$$
  The fitness of the *i*th beetle is defined as follows:
  7 $$f({\mathbf{x}}_{i} ) = 1 - A_{i}$$
Step 3: Obtain the searching behaviors of both right-hand and left-hand sides.
  In order to model the searching behavior, a random direction of beetle searching can be described as follows,
  8 $${\vec{\mathbf{b}}} = \frac{{{\mathbf{rv}}\left( {k,1} \right)}}{{eps + \left\| {{\mathbf{rv}}\left( {k,1} \right)} \right\|}}$$
  where ${\mathbf{rv}}\left( {m,1} \right)$ is a *m*-dimensional vector with random values between -1 and 1, *m* is the position's dimensions, here, *m* is set to 2, and $eps = 2^{{{ - }52}}$.
  The activities of the beetles’ antennae are imitated by the searching behaviors of both right-hand side and left-hand side, which are expressed as follows:
  9 $$\left\{ \begin{gathered} {\mathbf{x}}_{i,r} = {\mathbf{x}}_{i}^{t} + d^{t} {\vec{\mathbf{b}}} \hfill \\ {\mathbf{x}}_{i,l} = {\mathbf{x}}_{i}^{t} - d^{t} {\vec{\mathbf{b}}} \hfill \\ \end{gathered} \right.$$
  where ${\mathbf{x}}_{i,r}$ is the position which is lying in the searching area of the *i*th beetle's right-hand side, ${\mathbf{x}}_{i,l}$ is the position which is lying in the searching area of the *i*th beetle's left-hand side, and $d^{t}$ is the sensing length of antennae corresponding to the exploit ability at the *t*th time instant.
Step 4: Update the positions of the beetles.
  The iterative model is generated as Eq. (9) to associate with the odour detection by considering the searching behavior,
  10 $${\mathbf{x}}_{i}^{t} = {\mathbf{x}}_{i}^{t - 1} + \delta^{t} {\vec{\mathbf{b}}}sign\left( {f\left( {{\mathbf{x}}_{i,r} } \right) - f\left( {{\mathbf{x}}_{i,l} } \right)} \right)$$
  where $\delta^{t}$ is the step size of searching, and $sign\left( \cdot \right)$ is a sign function.
Step 5: Compare the fitness of ${\mathbf{x}}_{i}^{t}$ with the fitness of current best position of the *i*th beetle, if $f\left( {{\mathbf{x}}_{i}^{t} } \right) < f_{best}$, then $f_{best} = f\left( {{\mathbf{x}}_{i}^{t} } \right)$, ${\mathbf{x}}_{i,best} = {\mathbf{x}}_{i}^{t}$, where ${\mathbf{x}}_{i,best}$ is the current best position of the *i*th beetle, and $f_{i,best}$ is the fitness of current best position of the *i*th beetle.
Step 6: Update the antennae length $d$ and step size $\delta$ as follows,
  11 $$d^{t} = 0.95d^{{t{ - }1}} + r0$$
  12 $$\delta^{t} = 0.95\delta^{t - 1}$$
  where $r0$ is the constant.
Step 7: Repeat steps 2-6 until the stopping condition is met.
Step 8: Obtain the best position of the best fitness among all beetles, which is the optimal parameters of KELM.

### Fault diagnosis process of bearing based on GLCM and MBASA-based KELM

**Figure 3:**
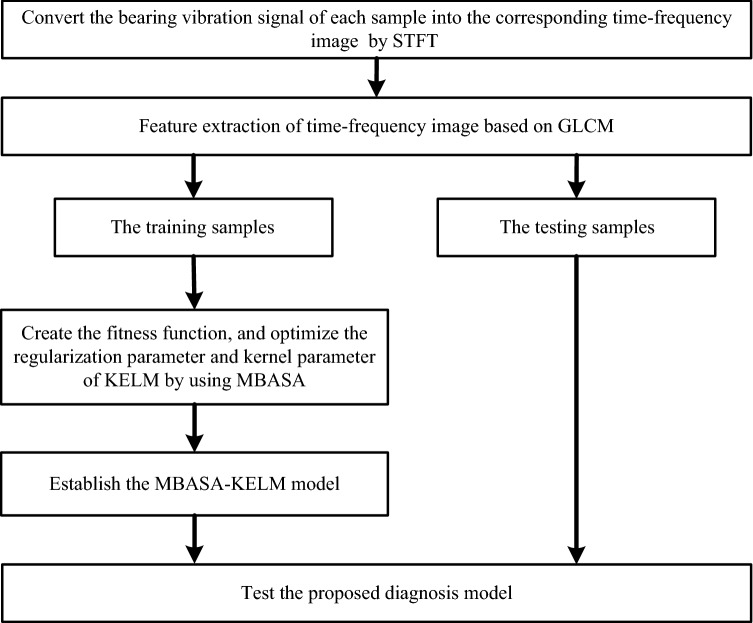
Fault diagnosis process of bearing based on GLCM and MBASA-based KELM.

Fault diagnosis process of bearing based on GLCM and MBASA-based KELM is given in Fig. 3. Firstly, convert the bearing vibration signal of each sample into the corresponding time–frequency image by STFT, and feature extraction of time–frequency image based on GLCM. Then, based on the training samples, create the fitness function, and optimize the regularization parameter and kernel parameter of KELM by using MBASA. Furthermore, establish the MBASA-KELM model by the optimized regularization parameter and kernel parameter of KELM. Finally, test the proposed diagnosis model.

## Experimental analysis

In the experiment, the data of the faults including inner race fault, outer race fault and ball fault with a damage size of 0.014 inches and the data of normal state under a load of 3HP are used, the sampling frequency is 48 kHz^19^. In the experiment, the 480 samples with 120 samples for each state are employed as the training samples, and the 320 samples with 80 samples for each state are employed as the testing samples.

The regularization parameter and kernel parameter of the KELM model are selected by MBASA. In MBASA, $d^{0}$ = 2, $\delta^{0}$ = 0.5,$r0 = 0.001$, and the range of the regularization parameter $C$ of the KELM model is [1,10000], and the range of Cauchy kernel parameter $\eta$ of the KELM model is [0.01,10].

LSSVM and KNN are respectively employed to compare with the proposed MBASA-KELM method. The regularization parameter and kernel parameter of the LSSVM model are selected by grid method, the range of the regularization parameter of LSSVM is $\{ 1,10,100,1000,10000\}$, and the range of Cauchy kernel parameter of LSSVM is $\{ 0.01,0.1,1,10\}$.The parameter *k* of the KNN model is selected by grid method, *k* is the natural number, and the range of the natural number *k* of KNN is [1,20].

**Figure 4:**
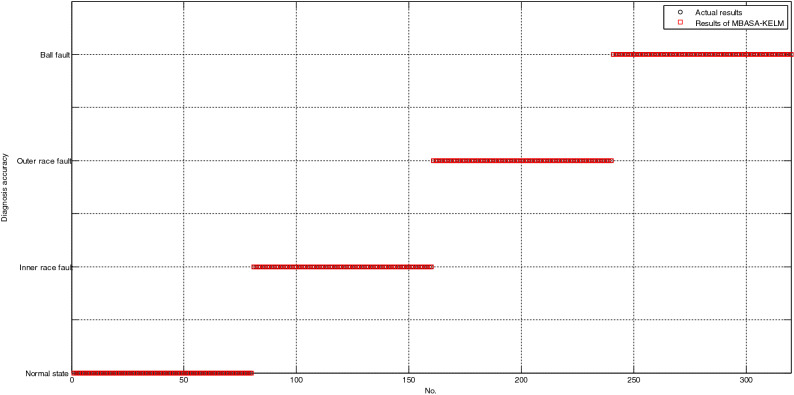
The diagnosis results of bearing based on MBASA-KELM.

**Figure 5:**
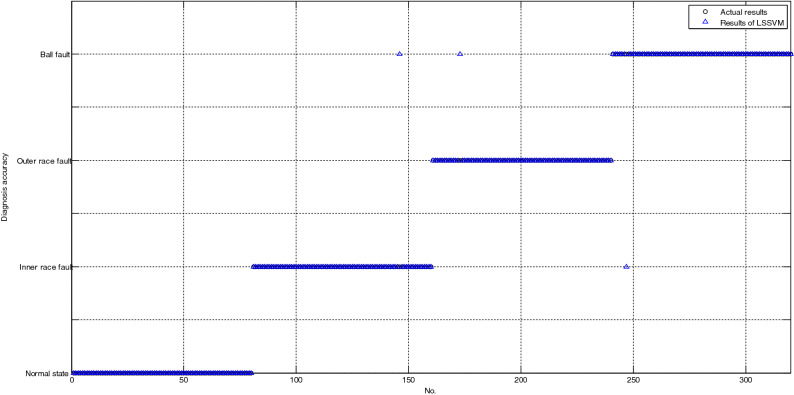
The diagnosis results of bearing based on LSSVM.

**Figure 6:**
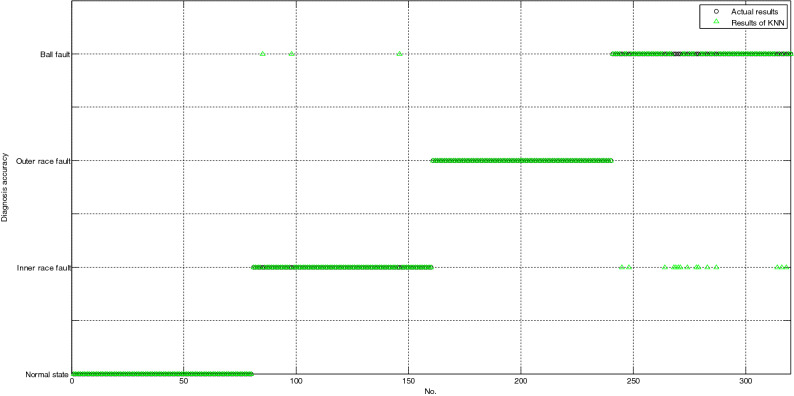
The diagnosis results of bearing based on KNN.

As shown in Fig. 4, all the testing samples are correctly classified based on MBASA-KELM; as shown in Fig. 5, the number of testing samples with incorrect diagnosis based on LSSVM is only 3; as shown in Fig. 6, the number of testing samples with incorrect diagnosis based on KNN is 18. Obviously, the diagnosis results of LSSVM for bearing are better than those of LSSVM, and KNN.

As shown in Table 1, the number of testing samples with correct diagnosis of MBASA-KELM is 320, and the diagnosis accuracy of MBASA-KELM is 100%, which demonstrates that feature extraction of time–frequency image based on GLCM can obtain excellent diagnosis results, and MBASA is helpful to select appropriate parameters of KELM; the number of testing samples with correct diagnosis of LSSVM is 317, and the diagnosis accuracy of LSSVM is 99.0625%; the number of testing samples with correct diagnosis of KNN is 302, and the diagnosis accuracy of KNN is 94.375%.It is indicated that MBASA-KELM has higher diagnosis accuracy for bearing than LSSVM, and KNN.

**Table 1:** The comparison of the diagnosis accuracies of bearing among MBASA-KELM, LSSVM, and KNN.

Diagnosis method	The number of testing samples with correct diagnosis	Diagnosis accuracy/%
MBASA-KELM	320	100
LSSVM	317	99.0625
KNN	302	94.375

## Conclusions

In this study, fault diagnosis method of bearing utilizing GLCM and MBASA-based KELM is presented, feature extraction of time–frequency image based on GLCM is proposed to extract the features of the bearing vibration signal, and MBASA-based KELM is presented to recognize the states of bearing.

STFT is used to convert the vibration signal into a time–frequency image, and feature extraction of time–frequency image based on GLCM is proposed in this study. GLCM is a texture feature extraction method for image pixel processing, and the basic principle of GLCM is to count the number of pairs of pixels whose distance is specified in the specified direction.

KELM with the kernel-based framework has better generalization than traditional extreme learning machine. MBASA employing multi-beetles is presented to select the regularization parameter and kernel parameter of KELM, which increases the possibility of obtaining the optimal parameters compared with one beetle antennae search. The experimental results demonstrate that MBASA is helpful to select appropriate parameters of KELM, and MBASA-KELM has better fault diagnosis ability for bearing than LSSVM, and KNN.

## Data Availability

The data used to support the findings of this study are available from the corresponding author upon request.
